# Supplementary material for: Content-rich biological network constructed by mining PubMed abstracts
Source: BMC Bioinformatics. 2004 Oct 8;5:147. doi: 10.1186/1471-2105-5-147 (PMC528731; doi:10.1186/1471-2105-5-147)
Supplement: Additional File 2 — The original results of the above study (non-essential files are deleted to keep the file size under the limit set by BMC bioinformatics). [file 1471-2105-5-147-S2.bz2 › chilibotAdditionalFile2/dip20/689ID9294424E18009/html/SECA_SECY.html]

 


 **SECA** and **SECY** 
  
Found 153 abstracts in PubMed, retrieved 20.  
 

 What does Google say? 
 PDF only 
| .edu only 

---

**Interactive relationship** (e.g. stimulation, inhibition, etc)

**Neutral relationship**- It is suggested that the C terminal  **SecY**  residues are required for  **SecA**  dependent translocation initiation.  Ref: 11914356 J Bacteriol, 2002
- The fifth and the sixth cytoplasmic regions C5 and C6 of  **SecY**  are important for the  **SecA**  driven preprotein translocation reaction.  Ref: 12351621 J Biol Chem, 2002
- CONCLUSIONS The Walker motif A regions of  **SecA**  are important for the  **SecA**   **SecY**  interaction that leads to the  **SecA**  conformational changes required for insertion into the SecYEG channel.  Ref: 11168585 Genes Cells, 2000
- In concert with SecB,  **SecA**  targets the precursors to  **SecY**  and energizes their translocation by its ATPase activity.  Ref: 11051763 Prog Nucleic Acid Res Mol Biol, 2001
- **SecY**  F286Y supports only a low  **SecA**  binding affinity, preprotein translocation and  **SecA**  translocation ATPase activity.  Ref: 11755523 FEBS Lett, 2002
- Thus, this mutant provides an excellent system for genetic analysis of the  **SecY**   **SecA**  interaction.  Ref: 11168585 Genes Cells, 2000
- The former class of mutations, presumably with specific effects on the  **SecY**   **SecA**  interaction, clustered in two regions close to the Walker motif A sequences of the two ATP binding domains.  Ref: 11168585 Genes Cells, 2000
- We introduced random mutations into a segment of Escherichia coli  **SecY**  within its cytoplasmic domain 5, which was shown previously to be important for the  **SecA**  dependent translocation activity.  Ref: 11309495 Proc Natl Acad Sci U S A, 2001
- In Escherichia coli, three different types of proteins engage the  **SecY**  translocon of the inner bacterial membrane for translocation or insertion 1 polytopic membrane proteins which prior to their insertion into the membrane are targeted to the translocon using the bacterial signal recognition particle SRP and its receptor, 2 secretory proteins which are targeted to and translocated across the  **SecY**  translocon in a  **SecA**  and SecB dependent reaction and 3 membrane proteins with large periplasmic domains, requiring SRP for targeting and  **SecA**  for the translocation of the periplasmic moiety.  Ref: 12682042 J Biol Chem< MedlineTA>J Biol Chem, 2001

**Non-interactive relationship** (e.g. studied together, co-existance, homology, etc.)

- One set is more similar to  **SecA**  and  **SecY**  of Escherichia coli.  Ref: 12010500 Mol Microbiol, 2002
- Genes encoding  **SecA**  and  **SecY**  proteins, essential components of the Sec protein translocation system, were cloned from onion yellows phytoplasma, an unculturable plant pathogenic bacterium.  Ref: 11551069 Mol Plant Microbe Interact, 2001
- Most bacterial species have a single copy of the genes encoding  **SecA**  and  **SecY** , which are essential for viability.  Ref: 12010500 Mol Microbiol, 2002
- However, Streptococcus gordonii strain M99 encodes  **SecA**  and  **SecY**  homologues that are not required for viability or for the translocation of most exported proteins.  Ref: 12010500 Mol Microbiol, 2002
- Pulse chase experiments showed that significant amount of wild type RBP was processed in a  **secY**  mutant in the presence of azide  **SecA**  inhibitor.  Ref: 12810088 Biochem Biophys Res Commun, 2003
- At least two other Gram positive species, Streptococcus pneumoniae and Staphylococcus aureus, encode two sets of  **SecA**  and  **SecY**  homologues.  Ref: 12010500 Mol Microbiol, 2002
- Once targeted to  **SecY** , ribosome associated nascent chains of the hybrid protein remain untranslocated unless  **SecA**  is present.  Ref: 11101515 EMBO J, 2000
- Previously, we have identified a cold sensitive  **secY**  mutation secY205, affecting the most carboxy terminal cytosolic domain, that did not allow an ATP dependent insertion of a  **SecA**  preprotein complex.  Ref: 11168585 Genes Cells, 2000
